# Association Studies in Clinical Pharmacogenetics

**DOI:** 10.3390/pharmaceutics15010113

**Published:** 2022-12-29

**Authors:** Pablo Zubiaur, Francisco Abad-Santos

**Affiliations:** 1 Clinical Pharmacology Department, Hospital Universitario de La Princesa, Instituto Teófilo Hernando, Instituto de Investigación Sanitaria La Princesa (IP), Universidad Autónoma de Madrid (UAM), 28029 Madrid, Spain; 2 Division of Clinical Pharmacology, Toxicology and Therapeutic Innovation, Children’s Mercy Research Institute, Kansas City, MO 64108, USA; 3 Centro de Investigación Biomédica en Red de Enfermedades Hepáticas y Digestivas (CIBERehd), Instituto de Salud Carlos III, 28200 Madrid, Spain

In recent times, the progress of Clinical Pharmacogenetics has been remarkable. Its implementation in the United States and Europe is gradually increasing. At the regulatory level, agencies such as the American Food and Drug Administration (FDA) or the European Medicines Agency (EMA) already incorporate genotyping indications in drug labels (e.g., siponimod-*CYP2C9* or abacavir-*HLA*-B*57:01). In addition, other consortia, such as the Clinical Pharmacogenetics Implementation Consortium (CPIC) or the Dutch Pharmacogenetics Working Group (DPWG), or national societies, such as the Spanish Society of Pharmacogenetics and Pharmacogenomics (SEFF), develop pharmacogenetic clinical guidelines with prescribing recommendations based on patient’s genotype. The development of these guidelines depends on the generation of pharmacogenetic evidence, which is collected by these entities. Eventually, associations with a high level of evidence are used to support dose adjustments or drug-change recommendations. The aim of this Special Issue was to compile sound works from any field of pharmacology that would increase the pharmacogenetic knowledge of drugs without previous clinical validation (e.g., *TYMS/ATIC/MTHFR* and methotrexate). Papers generating new and previously unpublished evidence were also considered. A total of 19 original articles that consider traditional association studies in clinical pharmacogenetics have been published, along with six systematic reviews and one research article with a more in vitro methodology approach. Most original research was published on cancer and neurodevelopmental and neurodegenerative disorders, followed by cardiovascular and autoimmune and inflammatory diseases.

The use of fluoropyrimidines is clearly related to pharmacogenetic biomarkers. The dihydropyrimidine dehydrogenase (DPD) enzyme, whose pharmacogenetic phenotype is well determined by genetic variations, metabolizes fluoropyrimidines extensively. *DPYD* decreased function or no function confers an intermediate or poor metabolizer phenotype. Prospective genotyping of the *DPYD* gene and dose reduction in intermediate metabolizers has proven to be beneficial for the patient and cost-effective, while these drugs may not be used in poor metabolizers [1]. However, a percentage of patients without high-risk genetic variants continue to experience fluoropyrimidine toxicity, both when administered in monotherapy and in combination. It is not surprising, therefore, that two of the published papers dealt with this issue. Novel variants in *DPYD* (rs367619008, rs200643089, and rs76387818) were related to severe drug toxicity [2], thus they could be considered potential biomarkers to test for the prescription of this family of drugs routinely and pre-emptively. Interestingly, *TYMS* rs11280056 was related to an increased risk for the development of neurotoxicity in FOLFIRINOX regimens [3]. This gene is part of the antimetabolite way and is involved in the pharmacodynamics of not only fluoropyrimidines, but also other cancer and immunomodulating drugs, such as methotrexate. TYMS inhibition by fluorodeoxyuridine monophosphate (fluoropyrimidine metabolite) prevents the synthesis of deoxythymidine monophosphate (dTPM), an essential compound for DNA synthesis, which causes cell death. *TYMS* rs11280056 was related to neurotoxicity occurrence. This variant causes an indel of AAGTTA in the 3′UTR region of the *TYMS* gene which could have an impact on protein function or expression, which could explain a pharmacodynamic alteration. The higher incidence of an adverse drug reaction suggests the interaction is more efficient when the deletion is present. Additional research is required to confirm the clinical relevance of this interaction.

Two works evaluated pharmacogenetic biomarkers for drugs prescribed to treat different types of breast cancer. A polymorphism in the upstream region of the *POLRMT* gene (rs62134260) was related to epirubicin-induced cardiac toxicity [4]. Furthermore, *FGFR1* copy number variations were related to drug response in *HER2*-positive breast cancer patients treated with trastuzumab and/or pertuzumab [5]. Both associations are novel and require confirmatory studies to consider their applicability in clinical practice. Given the prevalence and relevance of breast cancer, the personalized and individualized management of these patients may become a priority of our healthcare systems in the close future.

Hematopoietic stem cell transplantation is indicated for a variety of blood cancers, including different types of leukemia and myeloproliferative neoplasms among others. Drugs such as busulfan, a member of alkylating agents, may be used to prepare the patients who are going to receive hematopoietic stem cell transplantation. In patients undergoing hematopoietic stem-cell transplantation treated with busulfan, GSTA1*B promoter haplotype (defined by rs3957357 and rs3957356) was related to exposure variability [6]. This gene encodes for glutathione s-transferase (GST) A1 (GSTA1) isoform, the predominant GST isoform catalyzing busulfan metabolism (https://www.pharmgkb.org/pathway/PA165374494). The impact of *GSTA1* rs3957357 on busulfan metabolism was studied previously [7] but not as part of a haplotype, where the A allele was related to an increased drug clearance. In the work by Nguyen et al. [7], GSTA1*B promoter haplotype, which contains a T (A) nucleotide at position rs3957357, was related to a higher half-life, which corresponds to a lower clearance, which seems to contrast previous works. Additional research is warranted for the proper structural characterization of *GSTA1* genes and the impact of gene alleles on busulfan exposure and clinical outcomes. Finally, a comprehensive systematic review by Pinto-Merino et al. [8] was published, providing insights on the role of pharmacogenetics in the treatment of acute myeloid leukemia.

Continuing in the line of transplantation, several original research papers and reviews on the pharmacogenetics of tacrolimus were accepted in this Special Issue. As more novel associations, *MTHFR* rs1801133 in the donor and *MTHFR* rs1801131 in the recipient were related to survival rate variations in a 12-year follow-up period [9]. This gene is considered a very important pharmacogene (VIP) related to methotrexate toxicity. However, no clinically implementable information has been published to date. In fact, a recent DPWG guideline reiterated that there is no gene–drug interaction between *MTHFR* and methotrexate [10]. Moreover, in another systematic review on the pharmacogenetics of tacrolimus, authored by Lee et al., POR*28 was related to lower tacrolimus standardized concentrations [11]. However, to our knowledge, these pharmacogenes have not been structurally curated systematically to date (e.g., by institutions such as PharmVar). Additional research is required to characterize these genes, as clinical recommendations should be based on alleles (and the pharmacogenetic phenotype when available) rather than on individual variants. Finally, a traditional review authored by Brunet and Pastor-Anglada [12] provided a summary of currently available information regarding the influence of pharmacogenetics on the clinical outcome of patients treated with tacrolimus, where the role of CYP3A5 phenotype, tacrolimus trough levels and transporter genotypes is discussed.

Four works were accepted concerning neurodegenerative and neurodevelopmental disorders. Starting with the first type of disorders, *ABCB1* rs1045642, *ABCC2* rs2273697, and *SLC22A1* rs34059508 were related to variability in the exposure to rasagiline, a drug used for the management of patients with Parkinson’s disease (PD) [13]. To date, no pharmacogenetic biomarker has been proposed for rasagiline or any other drug prescribed for the treatment of this disease. Further research is warranted in this line so that a precision and personalized therapeutic approach can be offered to PD patients. Concerning neurodevelopmental disorders, *COMT* rs13306278, *ABCG2* rs2231142, CYP2B6, and CYP3A5 phenotypes were related to quetiapine exposure variability [14]; *NPY5R* rs11100494 was related to antipsychotic-induced weight gain. Antipsychotic drugs are prescribed for a variety of neurodevelopmental disorders, such as schizophrenia or bipolar disorder. A vast amount of works have been published to date [15,16,17], and some clinically relevant biomarkers are known (e.g., *CYP3A4*-quetiapine gene-drug interaction [18] or *CYP2D6*-aripiprazole [19]) for the personalized use of antipsychotic drugs. However, well-designed clinical trials or implementation studies are required to confirm the usefulness of most pharmacogenetic biomarkers described to date. In this line, Arranz et al. published an interesting implementation study where a pharmacogenetic intervention (prospective genotyping of *CYP1A2*, *CYP2C19*, *CYP2D6*, and *SLC6A4*) improved the clinical outcomes of treatment-resistant autistic spectrum disorder patients [20]. This confirms that not only is it important to generate new evidence and describe associations, but also to confirm their usefulness in routine practice.

Statins are prescribed for the treatment of hypercholesterolemia to reduce cardiovascular risk, and several pharmacogenetic biomarkers are well known to impact drug tolerability (i.e., *SLCO1B1*, *ABCG2*, and *CYP2C9*) [21]. Dagli-Hernandez et al. [22] observed a relationship between *ABCC1* rs45511401 and statins response in patients with familial hypercholesterolemia. Interestingly, Song et al. systematic review confirmed the association between *ABCG2* 421C>A (rs2231142) and rosuvastatin exposure [23]. Related to the cardiovascular system, two works evaluated direct oral anticoagulants (DOACs) tolerability. *AGT* rs5050 and *ACE* rs4353 were related to bleeding events [24], and so were *ABCB1* rs3842 and *APOB* rs13306198 [25]. Although DOACs improved the tolerability of vitamin K antagonists (such as warfarin acenocoumarin), pharmacogenetic biomarkers would potentially help reduce the incidence of bleeding/ischemic events in patients treated with this family of drugs.

Concerning autoimmune and inflammatory diseases, Chernov et al. observed a relationship between *ABCB1* rs1045642, rs1128503, and rs2032582 and cyclosporine response in patients with psoriasis [26]. Furthermore, Sainz et al. observed a relationship between *IL6R* rs4845625 and tocilizumab response in patients with rheumatoid arthritis [27]. In relation to the latter disease, Madrid-Paredes et al. [28] reviewed the genetic, epigenetic, and environmental factors related to the response of disease-modifying antirheumatic drugs.

For the treatment of postoperative pain, *C3orf20* rs12496846 was related to analgesic requirements (including opioids) [29]. Several pharmacogenetic guidelines are available for drugs used to manage pain, including nonsteroidal anti-inflammatory drugs [30] or opioids [31], which help reduce the risk for ADRs and ensure an effective pain control. Moreover, a systematic review on the pharmacogenetics of pain treatment with NSAIDs and antidepressants was published, authored by Zobdeh et al. [32].

Three additional associations were established: *ARMS2* rs10490924 and *CFH* rs1061170 were associated with ranibizumab response in patients with high myopia [33]; several variants in *FDPS* and *FNTA* were related to bisphosphonates response and in *CYP19A1* and *PDSS1* to selective estrogen receptor modulators (SERM) response in patients with osteoporosis [34]; finally, *ADCY9* rs879619, rs1042719, rs2531995, and *PDE4B* rs598961 were related to time to delivery variability in patients suffering preterm labor treated with ritodrine [35]. All the latter associations are rather novel and require confirmatory studies.

Finally, one study with a more in vitro methodology was accepted for publication, evaluating the impact of genetic variants and mRNA on *CYP1A2* function. Consistent with our association study with rasagiline [13], *CYP1A2* variants showed no relationship with CYP1A2 activity. This shows that the current definition of *CYP1A2* alleles is probably incorrect, and that priority should be given to the structural characterization of this gene. The proper characterization of pharmacogenes is a vital methodological step to later perform observational studies.

## References

[1] Amstutz U., Henricks L.M., Offer S.M., Barbarino J., Schellens J.H.M., Swen J.J., Klein T.E., McLeod H.L., Caudle K.E., Diasio R.B. (2018). Clinical Pharmacogenetics Implementation Consortium (CPIC) Guideline for Dihydropyrimidine Dehydrogenase Genotype and Fluoropyrimidine Dosing: 2017 Update. Clin. Pharmacol. Ther..

[2] Soria-Chacartegui P., Villapalos-García G., López-Fernández L.A., Navares-Gómez M., Mejía-Abril G., Abad-Santos F., Zubiaur P. (2021). Clinical Relevance of Novel Polymorphisms in the Dihydropyrimidine Dehydrogenase (DPYD) Gene in Patients with Severe Fluoropyrimidine Toxicity: A Spanish Case-Control Study. Pharmaceutics.

[3] Emelyanova M., Pokataev I., Shashkov I., Kopantseva E., Lyadov V., Heydarov R., Mikhailovich V. (2021). TYMS 3′-UTR Polymorphism: A Novel Association with FOLFIRINOX-Induced Neurotoxicity in Pancreatic Cancer Patients. Pharmaceutics.

[4] Velasco-Ruiz A., Nuñez-Torres R., Pita G., Wildiers H., Lambrechts D., Hatse S., Delombaerde D., Van Brussel T., Alonso M.R., Alvarez N. (2021). POLRMT as a Novel Susceptibility Gene for Cardiotoxicity in Epirubicin Treatment of Breast Cancer Patients. Pharmaceutics.

[5] Gaibar M., Novillo A., Romero-Lorca A., Malón D., Antón B., Moreno A., Fernández-Santander A. (2022). FGFR1 Amplification and Response to Neoadjuvant Anti-HER2 Treatment in Early HER2-Positive Breast Cancer. Pharmaceutics.

[6] Nguyen A.-H., Biswas M., Puangpetch A., Prommas S., Pakakasama S., Anurathapan U., Rachanakul J., Sukprasong R., Nuntharadtanaphong N., Jongjitsook N. (2022). Effect of GSTA1 Variants on Busulfan-Based Conditioning Regimen Prior to Allogenic Hematopoietic Stem-Cell Transplantation in Pediatric Asians. Pharmaceutics.

[7] Ten Brink M.H., van Bavel T., Swen J.J., van der Straaten T., Bredius R.G., Lankester A.C., Zwaveling J., Guchelaar H.-J. (2013). Effect of Genetic Variants *GSTA1* and *CYP39A1* and Age on Busulfan Clearance in Pediatric Patients Undergoing Hematopoietic Stem Cell Transplantation. Pharmacogenomics.

[8] Pinto-Merino Á., Labrador J., Zubiaur P., Alcaraz R., Herrero M.J., Montesinos P., Abad-Santos F., Saiz-Rodríguez M. (2022). Role of Pharmacogenetics in the Treatment of Acute Myeloid Leukemia: Systematic Review and Future Perspectives. Pharmaceutics.

[9] Sendra L., Olivera G.G., López-Andújar R., Serrano C., Rojas L.E., Montalvá E.M., Herrero M.J., Aliño S.F. (2022). Pharmacogene Variants Associated with Liver Transplant in a Twelve-Year Clinical Follow-Up. Pharmaceutics.

[10] Van der Pol K.H., Nijenhuis M., Soree B., de Boer-Veger N.J., Buunk A.M., Guchelaar H.-J., Risselada A., van Schaik R.H.N., Swen J.J., Touw D. (2022). Dutch Pharmacogenetics Working Group Guideline for the Gene-Drug Interaction of ABCG2, HLA-B and Allopurinol, and MTHFR, Folic Acid and Methotrexate. Eur. J. Hum. Genet..

[11] Lee D.-H., Lee H., Yoon H.-Y., Yee J., Gwak H.-S. (2022). Association of P450 Oxidoreductase Gene Polymorphism with Tacrolimus Pharmacokinetics in Renal Transplant Recipients: A Systematic Review and Meta-Analysis. Pharmaceutics.

[12] Brunet M., Pastor-Anglada M. (2022). Insights into the Pharmacogenetics of Tacrolimus Pharmacokinetics and Pharmacodynamics. Pharmaceutics.

[13] Zubiaur P., Matas M., Martín-Vílchez S., Soria-Chacartegui P., Villapalos-García G., Figueiredo-Tor L., Calleja S., Navares-Gómez M., de Miguel A., Novalbos J. (2022). Polymorphism of Drug Transporters, Rather Than Metabolizing Enzymes, Conditions the Pharmacokinetics of Rasagiline. Pharmaceutics.

[14] Zubiaur P., Fernández-Campos P., Navares-Gómez M., Soria-Chacartegui P., Villapalos-García G., Román M., Mejía-Abril G., Ochoa D., Abad-Santos F. (2021). Variants in COMT, CYP3A5, CYP2B6, and ABCG2 Alter Quetiapine Pharmacokinetics. Pharmaceutics.

[15] Soria-Chacartegui P., Villapalos-García G., Zubiaur P., Abad-Santos F., Koller D. (2021). Genetic Polymorphisms Associated With the Pharmacokinetics, Pharmacodynamics and Adverse Effects of Olanzapine, Aripiprazole and Risperidone. Front. Pharmacol..

[16] Ortega-Ruiz M., Soria-Chacartegui P., Villapalos-García G., Abad-Santos F., Zubiaur P. (2022). The Pharmacogenetics of Treatment with Quetiapine. Futur. Pharmacol..

[17] Zubiaur P., Soria-Chacartegui P., Villapalos-García G., Gordillo-Perdomo J.J., Abad-Santos F. (2021). The Pharmacogenetics of Treatment with Olanzapine. Pharmacogenomics.

[18] The Pharmacogenetics Working Group of the KNMP CYP3A4: Quetiapine (5991/5992).

[19] The Pharmacogenetics Working Group of the KNMP CYP2D6: Aripiprazole 1541/1542/1543.

[20] Arranz M.J., Salazar J., Bote V., Artigas-Baleri A., Serra-LLovich A., Triviño E., Roige J., Lombardia C., Cancino M., Hernandez M. (2022). Pharmacogenetic Interventions Improve the Clinical Outcome of Treatment-Resistant Autistic Spectrum Disorder Sufferers. Pharmaceutics.

[21] Cooper-DeHoff R.M., Niemi M., Ramsey L.B., Luzum J.A., Tarkiainen E.K., Straka R.J., Gong L., Tuteja S., Wilke R.A., Wadelius M. (2022). The Clinical Pharmacogenetics Implementation Consortium Guideline for *SLCO1B1*, *ABCG2*, and *CYP2C9* Genotypes and Statin-Associated Musculoskeletal Symptoms. Clin. Pharmacol. Ther..

[22] Dagli-Hernandez C., Borges J.B., Marçal E.d.S.R., de Freitas R.C.C., Mori A.A., Gonçalves R.M., Faludi A.A., de Oliveira V.F., Ferreira G.M., Bastos G.M. (2022). Genetic Variant ABCC1 Rs45511401 Is Associated with Increased Response to Statins in Patients with Familial Hypercholesterolemia. Pharmaceutics.

[23] Song Y., Lim H.-H., Yee J., Yoon H.-Y., Gwak H.-S. (2022). The Association between ABCG2 421C>A (Rs2231142) Polymorphism and Rosuvastatin Pharmacokinetics: A Systematic Review and Meta-Analysis. Pharmaceutics.

[24] Yee J., Song T.-J., Yoon H.-Y., Park J., Gwak H.-S. (2022). Genetic Factors of Renin–Angiotensin System Associated with Major Bleeding for Patients Treated with Direct Oral Anticoagulants. Pharmaceutics.

[25] Yoon H.-Y., Song T.-J., Yee J., Park J., Gwak H.-S. (2022). Association between Genetic Polymorphisms and Bleeding in Patients on Direct Oral Anticoagulants. Pharmaceutics.

[26] Chernov A., Kilina D., Smirnova T., Galimova E. (2022). Pharmacogenetic Study of the Impact of ABCB1 Single Nucleotide Polymorphisms on the Response to Cyclosporine in Psoriasis Patients. Pharmaceutics.

[27] Sainz L., Riera P., Moya P., Bernal S., Casademont J., Díaz-Torné C., Millán A.M., Park H.S., Lasa A., Corominas H. (2022). Role of IL6R Genetic Variants in Predicting Response to Tocilizumab in Patients with Rheumatoid Arthritis. Pharmaceutics.

[28] Madrid-Paredes A., Martín J., Márquez A. (2022). -Omic Approaches and Treatment Response in Rheumatoid Arthritis. Pharmaceutics.

[29] Nishizawa D., Nagashima M., Kasai S., Hasegawa J., Nakayama K., Ebata Y., Fukuda K., Ichinohe T., Hayashida M., Ikeda K. (2022). Associations between the C3orf20 Rs12496846 Polymorphism and Both Postoperative Analgesia after Orthognathic and Abdominal Surgeries and C3orf20 Gene Expression in the Brain. Pharmaceutics.

[30] Theken K.N., Lee C.R., Gong L., Caudle K.E., Formea C.M., Gaedigk A., Klein T.E., Agúndez J.A.G., Grosser T. (2020). Clinical Pharmacogenetics Implementation Consortium Guideline (CPIC) for *CYP2C9* and Nonsteroidal Anti-Inflammatory Drugs. Clin. Pharmacol. Ther..

[31] Crews K.R., Monte A.A., Huddart R., Caudle K.E., Kharasch E.D., Gaedigk A., Dunnenberger H.M., Leeder J.S., Callaghan J.T., Samer C.F. (2021). Clinical Pharmacogenetics Implementation Consortium Guideline for *CYP2D6*, *OPRM1*, and *COMT* Genotypes and Select Opioid Therapy. Clin. Pharmacol. Ther..

[32] Zobdeh F., Eremenko I.I., Akan M.A., Tarasov V.V., Chubarev V.N., Schiöth H.B., Mwinyi J. (2022). Pharmacogenetics and Pain Treatment with a Focus on Non-Steroidal Anti-Inflammatory Drugs (NSAIDs) and Antidepressants: A Systematic Review. Pharmaceutics.

[33] Blánquez-Martínez D., Díaz-Villamarín X., Antúnez-Rodríguez A., Pozo-Agundo A., Muñoz-Ávila J.I., Martínez-González L.J., Dávila-Fajardo C.L. (2021). Genetic Polymorphisms Affecting Ranibizumab Response in High Myopia Patients. Pharmaceutics.

[34] Del Real Á., Valero C., Olmos J.M., Hernández J.L., Riancho J.A. (2022). Pharmacogenetics of Osteoporosis: A Pathway Analysis of the Genetic Influence on the Effects of Antiresorptive Drugs. Pharmaceutics.

[35] Lee N., Yoon H.-Y., Park J.-Y., Kim Y.-J., Hwang H.-S., Yee J., Gwak H.-S. (2021). Association between ADCY9 Gene Polymorphisms and Ritodrine Treatment Outcomes in Patients with Preterm Labor. Pharmaceutics.

